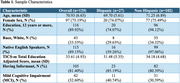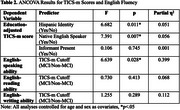# Impact of Sociodemographic and Linguistic Factors on TICS‐m Screening: Findings from the Remote‐CARE Study

**DOI:** 10.1002/alz70857_106205

**Published:** 2025-12-26

**Authors:** Nicole Sergeyev, Hannah Bodek, Chloe Moffitt, Hailey J. Andrews, Jack D. Cameron, Robert Lavin, Nelson A. Roque, Ali Ezzati, Richard B. Lipton, Laura A. Rabin

**Affiliations:** ^1^ Albert Einstein College of Medicine, Bronx, NY, USA; ^2^ Brooklyn College, City University of New York, Brooklyn, NY, USA; ^3^ The Pennsylvania State University, University Park, PA, USA; ^4^ CUNY Baccalaureate for Unique and Interdisciplinary Studies, City College of New York, New York, NY, USA; ^5^ Queens College, City University of New York, Queens, NY, USA; ^6^ The Graduate Center, CUNY, New York, NY, USA

## Abstract

**Background:**

The TICS‐m is a widely used, validated telephone‐based screening tool for assessing cognitive impairment in older adults. While educational adjustments are commonly applied to its scoring, demographic and linguistic factors may also impact performance on brief screenings. Additionally, social support, such as having an informant, may be associated with cognitive status. This study examines these factors in a diverse cohort of older adults recruited from the Bronx community through electronic health records (EHRs).

**Method:**

The Remote Cognitive Aging and Alzheimer's Disease REsearch (*R*‐CARE) study is an ongoing initiative aimed at validating a remote cognitive assessment toolbox for diverse, dementia‐free older adults, using a multimodal approach. Of the 1,496 individuals contacted, 158 agreed to participate in the phone screen, and total and domain TICS‐m scores were collected from 129 participants. Education adjusted TICS‐m scores (Gallo & Breitner, 1995) were compared across ethnic groups and informant status, while self‐reported English‐speaking ability was compared between participants scoring above and below the mild cognitive impairment cutoff (≤ 31). All analyses were conducted using an ANCOVA to adjust for demographic covariates (age and sex).

**Result:**

The sample had an average age of 70.93±6.65 and 75.2% were female (Table 1). Education adjusted TICS‐m scores were significantly higher in non‐Hispanic compared to Hispanic participants (*p* = 0.011) and in native English‐speakers compared to non‐native speakers (*p* = 0.007). Among non‐native speakers, self‐reported English‐speaking ability was significantly higher in those scoring above the cutoff compared to those scoring below (*p* = 0.028), after controlling for covariates (Table 2). No significant differences were observed in self‐reported fluency domains of reading and writing. Individuals with informants exhibited numerically higher overall and domain scores on the TICS‐m scores although these differences did not reach statistical significance. Findings were consistent for education‐adjusted and unadjusted TICS‐m scores.

**Conclusion:**

This study highlights how sociodemographic and linguistic factors influence TICS‐m performance, with lower scores observed among Hispanic and non‐native English speakers, even after adjusting for education. These findings underscore the need for tailored cognitive screening approaches that account for linguistic and cultural differences to ensure accurate assessment of cognitive status. Follow‐up assessments will explore how TICS‐m performance predicts cognitive outcomes.